# Fluorescein-guided surgery for the resection of pilocytic astrocytomas: A multicentric retrospective study

**DOI:** 10.3389/fonc.2022.943085

**Published:** 2022-08-09

**Authors:** Jacopo Falco, Julius Höhne, Morgan Broggi, Emanuele Rubiu, Francesco Restelli, Ignazio G. Vetrano, Marco Schiariti, Elio Mazzapicchi, Giulio Bonomo, Paolo Ferroli, Karl-Michael Schebesch, Francesco Acerbi

**Affiliations:** ^1^ Department of Neurosurgery, Fondazione IRCCS Istituto Neurologico Carlo Besta, Milan, Italy; ^2^ Department of Neurosurgery, Department of Neurosurgery, University Medical Center Regensburg, Regensburg, Germany

**Keywords:** pilocytic astrocytoma, central nervous system tumors, sodium fluorescein, neuro-oncology, YELLOW 560 filter, fluorescence-guided neurosurgery

## Abstract

**Objective:**

Pilocytic astrocytomas (PAs) are relatively benign tumors, usually enhancing on post-contrast MRI and often characterized by a mural nodule within a cystic component. Surgical resection represents the mainstay of treatment, and extent of resection (EOR) is associated with improved survival. In this study, we analyzed the effect of sodium fluorescein (SF) on the visualization and resection of these circumscribed astrocytic gliomas.

**Methods:**

Surgical databases at two neurosurgical departments (Fondazione IRCCS Istituto Neurologico Carlo Besta, Milan, Italy and Department of Neurosurgery, University Medical Center Regensburg, Regensburg, Germany) were retrospectively reviewed to identify the cohort of patients with pilocytic astrocytoma who had undergone fluorescein-guided tumor resection at any of the centers between March 2016 and February 2022. SF was intravenously injected (5 mg/kg) immediately after the induction of general anesthesia. Tumors were removed using a microsurgical technique with the YELLOW 560 filter (Carl Zeiss Meditec, Oberkochen, Germany).

**Results:**

Forty-four patients (25 males and 19 females; 26 pediatric patients, mean age of 9.77 years, range 2 to 17 years; and 18 adult patients, mean age of 34.39 years, range 18 to 58 years) underwent fluorescein-guided surgery. No side effects related to SF occurred. In all tumors, contrast enhancement on preoperative MRI was correlated with intense, heterogeneous yellow fluorescence with bright fluorescent cystic fluid. Fluorescein was considered helpful in distinguishing tumors from viable tissue in all cases except three patients due to faint fluorescein enhancement. Biopsy was intended in two operations, and partial resection was intended in three operations. Gross total resection was achieved in 24 cases out of 39 patients scheduled for tumor removal (61.54%), in five cases a minimal residual volume was highlighted by postoperative MRI despite the intraoperative subjective evaluation of complete tumor removal (12.82%); in the other 10 cases, the resection was subtotal with fluorescent residual spots to avoid neurological worsening (25.64%).

**Conclusions:**

The use of SF is a valuable method for safe fluorescence-guided tumor resection. Our data showed a positive effect of fluorescein-guided surgery on intraoperative visualization during resection of Pas, suggesting a possible role in improving the extent of resection of these lesions.

## Introduction

Pilocytic astrocytomas (PAs) are tumors that are often described as slow-growing lesions (1–3) with an indolent course. These astrocytic lesions present a peculiar epidemiological distribution as they are considered the most common glioma subtype among children, with a peak incidence in the first two decades of life, presenting a significantly lower occurrence in adults (4, 5). According to the 2016 (6) and 2021 (7) World Health Organization (WHO) tumor classification, pilocytic astrocytomas are classified as grade I and grade 1 tumors, respectively, and they are associated with an excellent prognosis, with a 10-year survival rate of more than 95% (8, 9). Notwithstanding the favorable clinical course of most PAs, a small subset of tumors is associated with rapid recurrence, especially for high proliferative tumor cells and unfavorable mutations (8, 10).

Those PAs present at MRI with well-circumscribed margins usually have both solid (predominant) and cystic components, with inhomogeneous contrast enhancement due to blood–brain barrier (BBB) disruption (1, 11).

The curative potential of surgical resection (2, 9, 12) is extremely affected by the location of the lesion and the difficulty, during intraoperative visualization, in discriminating the tumor margins; in particular, the hypothalamus or opticochiasmatic region involvement can affect the prognosis; indeed, for these tumors, surgical resectability can sometimes be impossible; therefore, a chemotherapy approach is advisable after biopsy or debulking surgery. In this perspective, the application of new technical tools aimed at improving the extent of resection (EOR) could be beneficial. Patients are usually followed up by regular imaging as re-resection is advised at recurrence (12). Because of its long-term benign course, radiation therapy is generally considered in selected cases (8); palliative care with chemotherapy, radiation therapy, or both is an option for unresectable and multiple recurrent tumors (13).

Sodium fluorescein (SF) is a dye that, when intravenously injected, has the peculiar characteristic of accumulation in cerebral areas, presenting damage to the BBB (14, 15). The use of a dedicated filter in the surgical microscope, with a specific wavelength for fluorescein (540–690 nm), allows one to improve the tumor–brain discrimination intraoperatively, reducing the dosage needed to obtain this effect (16–18). In particular, this has been shown to be associated with better tumor identification and resection in different tumors of the central nervous system (CNS), such as high-grade gliomas (19, 20), gangliogliomas (21), cerebral metastasis (22), and primary CNS lymphomas (23), both in adult and pediatric populations (24).

In July 2015, based on preliminary scientific results from different studies published in the international literature (25, 26), including a prospective phase II trial from our group, the Italian Medicine Agency (AIFA) has extended the indications for the usage of fluorescein molecule (https://www.gazzettaufficiale.it/atto/serie_generale/caricaDettaglioAtto/originario;jsessionid=izVcTOmnjOzfNRjjw56kAA:.ntc-as2-guri2b?atto.dataPubblicazioneGazzetta=2015-07-22&atto.codiceRedazionale=15A05620&elenco30giorni=false). According to this determination, the intravenous (i.v.) injection of SF as a neurosurgical tracer during oncological procedures for aggressive tumors of the CNS is approved and its cost is totally reimbursed by the Italian National Health System (20). In particular, a low dose (5 mg/kg) of fluorescein is i.v. administered at the end of patient intubation (around 1 h before dural opening); patients of any age and gender can be operated by means of SF according to this standardized protocol according to AIFA determination.

Due to the characteristics of MRI contrast uptake in PAs, the use of SF as a fluorescent tracer could allow better intraoperative discrimination of the tumoral tissue even in this tumor subtype, with beneficial effects during surgical resection (4). The foundation of our study rests upon the intrinsic staining potential of fluorescein and the radiological features of PAs, characterized by an intense contrast-enhanced pattern. The rational is the usage of fluorescein molecule to selectively stain the contrast-enhanced areas of PAs to improve the intraoperative visualization of the solid portion of the tumor. In the presented paper, we performed a retrospective multicentric analysis of the contribution of fluorescein-guided technique for the removal of PAs, aiming to show the advantages and valuable role of fluorescein in the visualization and resection of pilocytic astrocytomas.

## Methods

### Patients and inclusion criteria

In this study, we systematically and retrospectively reviewed surgical and neuropathological databases at two neurosurgical departments (Fondazione IRCCS Istituto Neurologico Carlo Besta, Milan, Italy and Department of Neurosurgery, University Medical Center Regensburg, Regensburg, Germany) to identify the cohort of patients affected by pilocytic astrocytoma who had undergone fluorescein-guided tumor resection at any center between March 2016 and February 2022. Inclusion criteria were informed consent about the use of SF, histopathologically confirmed PAs according to 2016 or 2021 WHO CNS tumor classification, and no known allergy to fluorescein. The surgical databases of the two Institution, from which the patient data were retrieved, were approved by the respective Institutional Review Boards. Additionally, the cohort of patients operated at the Fondazione IRCCS Istituto Neurologico Carlo Besta were enrolled in a prospective observational study on the use of SF for contrast enhancing the resection of CNS tumors (20), the FLUOCERTUM study (FLUOrescein in CERebral TUMors), also approved by the Institutional Review Board.

### Clinical and radiological management

Preoperative assessment included physical and neurological examination (Karnofsky Performance Status [KPS] (27) in adult patients and Lansky Play-Performance status [LPS] (28) in pediatric ones), laboratory test results, preoperative contrast-enhanced MRI for neuronavigation. In preoperative MRI, patients were categorized based on their preoperative contrast enhancement characteristics. To evaluate the EOR, a volumetric MRI was performed for each patient within 72 h after surgery; in particular, to calculate the residual pathological volume, the hyperintense alterations in volumetric basal T1 acquisitions were subtracted from the volume of hyperintense tissue in post-contrast volumetric T1 images, to avoid the incidental inclusion of blood or blood products (29). The EOR was calculated as a percentage of tumor resection based on early contrast-enhanced postoperative MRI; according to the entity of resection, we distinguished four main categories: gross total resection (GTR, EOR 100%), sub-total resection (STR, with an EOR of 90%–100%), partial resection (PR, 30%–90%), and biopsy. The postoperative clinical evaluation included a standard neurological examination as above, a laboratory test (kidney function), and the exclusion of the occurrence of any side effect related to fluorescein injection. Clinical and neuroradiological long-term follow-up were performed for the postoperative period as part of normal clinical practice, including a telephonic interview.

### Surgical protocol

The standardized surgical protocol of the fluorescein-guided technique, as already described in previous papers (16, 17, 20, 25, 26), is based on i.v. SF (Monico S.p.A., Venice, Italy and Alcon Pharma, Freiburg im Breisgau, Germany) injection at a standard dose of 5mg/kg, using a central or peripheral venous line, immediately upon completion of the induction of general anesthesia. Surgery was performed with the aid of a surgical microscope equipped with an integrated fluorescent filter tailored to the excitation and emission wavelengths of sodium fluorescein (YELLOW 560—Pentero 900 and Kinevo; Carl Zeiss Meditec, Oberkochen, Germany). During resection, the microscope could be switched alternatively from fluorescent to white-light illumination; neuronavigation, intraoperative contrast-enhanced ultrasounds, or other tools could be used according to the preference of the surgeon. Intraoperative neurophysiological monitoring was used in tumors located adjacent to eloquent areas. The tumors were removed in an inside-out fashion until all fluorescent tissue was removed, as considered feasible by the surgeon.

### Intraoperative fluorescence characteristics and side effects

Fluorescence intensity was graded by the surgeon as intense, inhomogeneous or homogeneous, moderate, slight or absent; surgeons were also asked to classify the use of SF per procedure as helpful, partially helpful, unhelpful or not essential to achieve surgical aims. Furthermore, medical reports were evaluated for any possible adverse effect or allergic reaction to fluorescein administration.

### Statistical analysis

The sample was described by means of the usual descriptive statistics: mean, median, and standard deviation for continuous variables and proportions for categorical ones. PRISM software for the Macintosh was used for the statistical analysis.

## Results

### Patients population

We enrolled 44 patients (25 males and 19 females; 26 pediatric patients, mean age 9.77 years, range 2 to 17 years; and 18 adult patients, mean age 34.39 years, range 18 to 58 years) affected by pilocytic astrocytomas.

**Figure 1:**
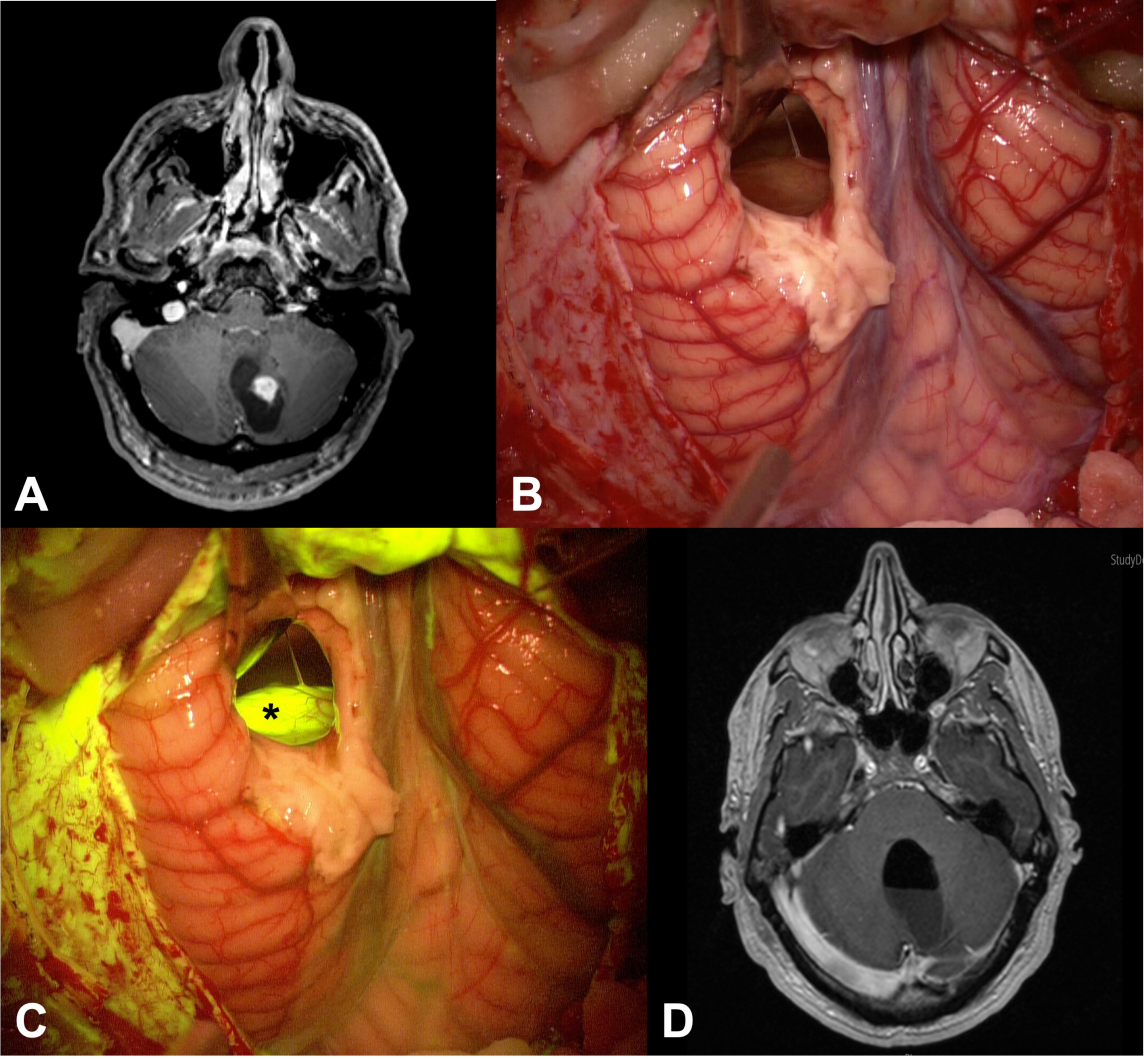
**(A)** Preoperative axial post-contrast T1 MRI of patient n.26 showed a left cerebellar lesion with its enhancing solid component and a large cystic component. **(B, C)** Intraoperative images: after cystic distension through a small corticectomy, the solid part of the tumor was exposed **(B)**, appearing as a greyish tissue; this tissue appeared more evident when the YELLOW 560 filter was activated (asterisk in **(C)**), as a bright and intense fluorescence. **(D)** Postoperative axial post-contrast T1 MRI demonstrated complete tumor removal of the lesion.

**Figure 2:**
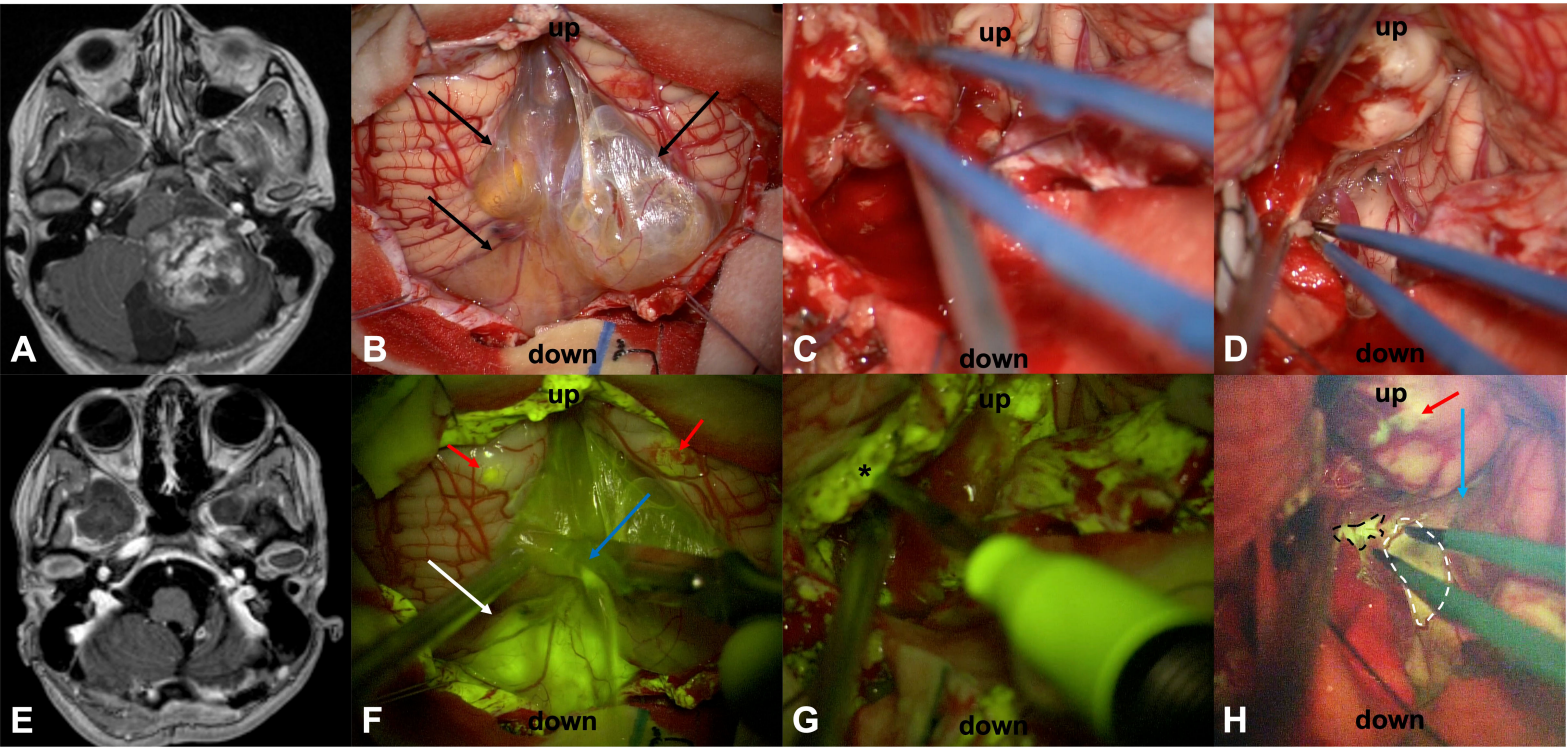
Preoperative T1 with contrast axial scan **(A)** in a 14-year-old female (patient n.20) shows a large posterior cranial fossa pilocytic astrocytoma with a subtotal resection, as detectable by postoperative post-contrast T1 MRI in **(E)**. Surgery was performed in the sitting position. After dural opening, it was possible to appreciate a partially cystic lesion (arrows in **(B)**) that was better highlighted after activation of the YELLOW 560 filter **(F)**, as a multilobulated cyst with a bright fluid (blue arrow) and an inferior vermis nodular component with an intense fluorescein enhancement (white arrow). Two small cortical areas of unspecific enhancement due to cerebellar manipulation during the initial phase of dural opening were visible as fluorescent spots (red arrows in **(F)**): this could happen because the damage to the cortex has been performed closer to fluorescein i.v. injection when a significant amount of fluorescein molecules is still present in blood circulation. The tumor was removed with a cavitron ultrasound aspirator, showing a bleeding, friable tissue **(C)** with inhomogeneous fluorescein enhancement after YELLOW 560 activation (asterisk in **(G)**). SF resulted particularly important to discriminate pathological fluorescent tissue **(dotted lines)** from normal peritumoral parenchyma (blue arrow) at tumor borders **(D**–**H)**: minimal fluorescent tissue was left where the infiltration of the left lateral recess (black dotted line) and, less intensely, on the left part of the floor of the IV ventricle (white dotted line) made surgical resection hazardous for neurological functioning. The fact that the same minimal area of infiltration was not visible at postoperative MRI is related to the molecular weight of fluorescein, which is slightly lower than that of the contrast medium, that allows for a better delineation of BBB damage even in areas where contrast enhancement was not present. The unspecific enhancement due to cerebellar surgical manipulation at the beginning of the surgery was still visible with the YELLOW 560 filter activated (red arrow in **H**).

All the patients presented with variable contrast enhancement patterns in the preoperative MRI: PAs mostly showed a solid, highly enhancing component (the mural nodule) with a large cyst (**Figure 1**); furthermore, several other contrast enhancement patterns were observed, such as homogeneous and intense contrast uptake or peripheral, heterogeneous contrast enhancement with central cystic-necrotic areas (**Figure 2**).

Data on the clinical condition at admission, discharge, and follow-up and intraoperative findings were available for all patients (**Table 1**).

**Table 1:** Characteristics of the patients and main results.

Nr.	Age	Gender	DOS	Clinical presentation	KPS/LPS pre-op	Tumor location	Fluorescence intensity	Intra-op side effects	Surgeon’s opinion	Residual fluorescence (Y/N - explanation)	EOR (GTR, intended STR, unintended STR, PR-debulking, biopsy)	Histology	KPS/LPS post-op	FU (Timing)	FU (Radiological)	FU (KPS/LPS)
**1**	6	F	08/03/16	headache, visual disturbance	80	hypothalamus, opticochiasmatic	moderate	no	helpful	N	unintended STR	pilocytic astrocytoma (WHO I)	80	5.2020	tumor recurrency (→ CHT)	70
**2**	12	M	12/09/16	left motor hemisyndrome	60	right thalamo-mesencephalic	moderate	no	helpful	Y—incomplete resection of the fluorescent component: infiltration of the basal ganglia and thalamus	intended STR	pilocytic astrocytoma (WHO I)	70	3.2021	stable tumor	90
**3**	18	M	22/12/16	nausea and altered color perception	90	right temporo-mesial, left frontal	homogeneously intense	no	helpful	N	GTR	pilocytic astrocytoma (WHO I)	100	8.2021	tumor free	100
**4**	3	F	10/03/17	NF1; right hemisyndrome	80	left basal ganglia	moderate	no	helpful	N	unintended STR	pilocytic astrocytoma (WHO I)	90	1.2022	stable tumor	90
**5**	6	F	16/03/17	functional impairment right superior limb, repetitive movements right hand and dystonic posture, aphasia	80	left basal ganglia and lateral ventricle	moderate (bright cystic fluid)	no	helpful	N	unintended STR	pilocytic astrocytoma (WHO I)	70	12.2019	progression disease (→ ReSurgery—25)	90
**6**	7	M	28/03/17	hypotrophy and hyposthenia right inferior limb	70	D10-L1	slight	no	partially helpful	Y – biopsy	Biopsy	pilocytic astrocytoma (WHO I)	70	10.2021	stable tumor	60
**7**	18	M	23/03/18	headache, dysphagia, ataxia	60	midbrain, pons, right cerebellar	heterogeneously intense	no	helpful	Y – incomplete resection of the fluorescent component: tenacious adhesions to cranial nerves and brainstem	intended STR	pilocytic astrocytoma (WHO I)	60	2.2022	progression disease (–> ReSurgery—44)	50
**8**	14	M	08/05/18	headache	100	left temporal	homogeneously intense	no	helpful	N	GTR	pilocytic astrocytoma (WHO I)	100	1.2022	tumor free	100
**9**	57	F	15/06/18	cerebellar syndrome	60	posterior cranial fossa	heterogeneously intense	no	helpful	Y – incomplete resection of the fluorescent component: infiltration of the right cerebellar peduncle	intended STR	pilocytic astrocytoma (WHO I)	70	10.2019	progression disease (→ CHT/RT)	80
**10**	44	F	15/06/18	seizure	90	left frontal	homogeneously intense	no	helpful	Y – biopsy	Biopsy	pilocytic astrocytoma (WHO I)	90	4.2020	stable tumor	90
**11**	17	M	12/07/18	seizure	90	left parieto-occipital	homogeneously intense (bright cystic fluid)	no	helpful	N	GTR	pilocytic astrocytoma (WHO I)	90	1.2022	tumor free	100
**12**	20	M	02/08/18	convulsions, speech arrest	90	left temporo-insular	moderate	no	helpful	N	GTR	pilocytic astrocytoma (WHO I)	90	4.2021	tumor free	100
**13**	56	M	11/09/18	nausea, vomiting	100	IV ventricle	moderate	no	helpful	N	GTR	pilocytic astrocytoma (WHO I)	100	11.2021	tumor free	100
**14**	15	M	13/09/18	NF1	100	left lateral ventricle	homogeneously intense	no	helpful	N	GTR	pilocytic astrocytoma (WHO I)	100	12.2021	tumor free	100
**15**	2	F	14/09/18	vertigo, squint, vomiting	70	left cerebllar hemisphere	homogeneously intense	no	helpful	N	GTR	pilocytic astrocytoma (WHO I)	100	5.2021	tumor free	100
**16**	11	M	01/10/18	triventricular hydrocephalus	90	pineal gland, dorsal midbrain	heterogeneously intense	no	helpful	Y – incomplete resection of the fluorescent component: infiltration of the midbrain	intended STR	pilocytic astrocytoma (WHO I)	90	11.2021	stable tumor	100
**17**	14	M	08/10/18	headache, nausea and sickness	90	right cerebellar	heterogeneously intense	no	helpful	N	GTR	pilocytic astrocytoma (WHO I)	90	6.2021	tumor free	100
**18**	18	M	06/12/18	seizure	90	right temporal	moderate	no	partial helpful	N	unintended STR	pilocytic astrocytoma (WHO I)	90	3.2022	stable tumor	90
**19**	18	M	13/12/18	headache	100	right temporal	moderate	no	helpful	N	GTR	pilocytic astrocytoma (WHO I)	100	11.2020	tumor free	100
**20**	14	F	24/01/19	headache, ideomotor impairment	60	posterior cranial fossa	heterogeneously intense (bright cystic fluid)	no	helpful	Y—incomplete resection of the fluorescent component: infiltration of the IV ventricle	intended STR	pilocytic astrocytoma (WHO I)	80	5.2021	stable tumor	90
**21**	11	F	15/04/19	vertigo, headache	100	IV ventricle	homogeneously intense	no	helpful	N	GTR	pilocytic astrocytoma (WHO I)	100	7.2019	tumor free	100
**22**	6	F	07/06/19	vertigo, visual deficits, vomiting	80	right cerebellar hemisphere	homogeneously intense	no	helpful	N	GTR	pilocytic astrocytoma (WHO I)	90	11.2021	tumor free	100
**23**	37	F	27/09/19	bilateral cervicodorsalgia	70	D2-D3	moderate	no	helpful	Y – incomplete resection of the fluorescent component: infiltrative behavior with IONM positive response	intended STR	pilocytic astrocytoma (WHO I)	50	3.2022	stable tumor	60
**24**	10	M	20/11/19	headache and diplopia	90	posterior cranial fossa	heterogeneously intense	no	helpful	N	GTR	pilocytic astrocytoma (WHO I)	70	2.2022	tumor free	80
25 - 5*	9	F	16/12/19	FU pat.5 (functional impairment right superior limb, aphasia)	90	left temporal and lateral ventricle	homogeneously intense	no	helpful	Y—incomplete resection of the fluorescent component: infiltration of the left CST	intended STR	pilocytic astrocytoma (WHO I)	90	2.2022	stable tumor	90
**26**	21	F	24/02/20	diplopia, headache, postural instability	80	left cerebellar	homogeneously intense (bright cystic fluid)	no	helpful	N	GTR	pilocytic astrocytoma (WHO I)	80	9.2021	tumor free	90
**27**	13	M	02/04/20	seizure	90	right frontal	moderate	no	helpful	N	GTR	pilocytic astrocytoma (WHO I)	90	11.2021	tumor free	100
**28**	5	M	26/06/20	left hemiparesis, visual loss	60	hypothalamus, opticochiasmatic	heterogeneously intense	no	helpful	Y—incomplete resection of the fluorescent component: infiltration of the optic chiasm	PR-debulking	pilocytic astrocytoma (WHO I)	60	1.2022	stable tumor	70
**29**	9	M	04/08/20	left VII c.n. deficit, ataxia	70	right thalamo-mesencephalic	moderate	no	helpful	N	GTR	pilocytic astrocytoma (WHO I)	80	10.2021	tumor free	90
**30**	49	F	14/10/20	headache	70	left hippocampal	homogeneously intense	no	helpful	N	GTR	pilocytic astrocytoma (WHO I)	70	6.2021	tumor free	70
**31**	6	M	04/11/20	optic neuropathy	80	hypothalamus, opticochiasmatic	homogeneously intense	no	helpful	Y—incomplete resection of the fluorescent component: infiltration of the optic chiasm	intended STR	pilocytic astrocytoma (WHO I)	80	11.2021	stable tumor	80
**32**	54	F	26/11/20	NF1	90	left frontal	slight	no	not helpful	N	GTR	pilocytic astrocytoma (WHO I)	90	1.2022	tumor free	90
**33**	58	M	09/02/21	amnesia	80	III ventricle	moderate	no	helpful	Y—incomplete resection of the fluorescent component: infiltration of the hypothalamus	intended STR	pilocytic astrocytoma (WHO I)	70	10.2021	stable tumor	80
**34**	26	F	26/02/21	radiological FU in pre-existing encelphalitis disseminata	100	posterior callosal body, right lateral ventricle	homogeneously intense	no	helpful	N	unintended STR	pilocytic astrocytoma (WHO I)	100	1.2022	progression disease	100
**35**	8	M	04/06/21	headache, nausea and vomiting	80	cerebellar vermis	homogeneously intense	no	helpful	N	GTR	pilocytic astrocytoma (WHO I)	90	10.2021	tumor free	90
**36**	16	M	15/06/21	ideomotor impairment, right hemisoma weakness	90	left cerebellar peduncle	homogeneously intense (bright cystic fluid)	no	helpful	N	GTR	pilocytic astrocytoma (WHO I)	90	10.2021	tumor free	100
**37**	13	M	13/09/21	nausea, vomiting	70	right temporal parietal	moderate	no	helpful	N	GTR	pilocytic astrocytoma (WHO I)	80	3.2022	recurrent tumor	100
**38**	15	F	29/10/21	headache, right hemiparesis	60	left temporal	homogeneously intense	no	helpful	N	GTR	pilocytic astrocytoma (WHO I)	90	3.2022	tumor free	90
**39**	23	M	13/12/21	nausea, ataxia, nystagmus	80	IV ventricle	heterogeneously intense (bright cystic fluid)	no	helpful	Y—incomplete resection of the fluorescent component: infiltration of the IV ventricle and right Luschka foramen	intended STR	pilocytic astrocytoma (WHO I)	40	\	\	\
**40**	53	F	16/12/21	ptosis, double vision	100	right frontal temporal	homogeneously intense	no	helpful	N	GTR	pilocytic astrocytoma (WHO I)	80	2.2022	tumor free	80
**41**	6	M	12/01/22	left hemiparesis, hydrocephalus	60	hypothalamus, diencephalon	heterogeneously intense (bright cystic fluid)	no	helpful	Y—incomplete resection of the fluorescent component: tenacious adhesions to eloquent areas	PR-debulking	pilocytic astrocytoma (WHO 1)	60	\	\	\
**42**	27	F	27/01/22	headache	100	right fronto-parietal	homogeneously intense	no	helpful	N	GTR	pilocytic astrocytoma (WHO 1)	100	\	\	\
**43**	6	F	01/02/22	vertigo, nausea, vomiting	90	IV ventricle	homogeneously intense	no	helpful	N	GTR	pilocytic astrocytoma (WHO 1)	100	\	\	\
44 - 7*	22	M	24/02/22	FU pat.7 (dysphagia, dysphonia, dysarthria and ataxia)	50	posterior cranial fossa	heterogeneously intense (bright cystic fluid)	no	helpful	Y—incomplete resection of the fluorescent component: tenacious adhesions to eloquent areas	PR-debulking	pilocytic astrocytoma (WHO 1)	40	\	\	\

### Intraoperative fluorescence characteristics and surgeon’s opinion

Homogeneously intense fluorescent staining was reported in 19/44 cases (43.18%) whereas an inhomogeneous and intense fluorescence enhancement was described in 10 patients (22.73%); a moderate fluorescence was detected in 13/44 cases (29.55%) while in only two patients (4.55%) pathological tissue appeared slightly fluorescent (**Table 1**). In eight multicystic tumors (18.18%), independently from the specific fluorescein enhancement, we observed a bright fluorescent cystic fluid (**Figures 1**, **2**). Preoperative corticosteroid therapy did not affect fluorescence characteristics.

In all cases but three, intraoperative fluorescence was deemed helpful (93.18%) in achieving a complete resection using a better delineation of the borders of the tumor tissue from the healthy parenchyma as compared with the conventional microsurgical technique using white-light illumination. In two cases (4.55%), SF was considered partially helpful in identifying the tumor (**Table 1**): in fact, in one patient the fluorescence enhancement appeared moderate with tenuous SF uptake at tumor borders (Pat.18) whereas in the other one, SF, whose enhancement was slight, was used along with intraoperative ultrasound to identify the biopsy target (Pat.6). Finally, in one patient (2.27%), the tumor was slightly fluorescent under a YELLOW 560 filter, so SF was judged unhelpful in facilitating tumor resection (Pat.32). Intraoperative fluorescence corresponded to preoperative MRI documented contrast enhancement.

No technical difficulties regarding the use of the microscope filter nor switching between white and yellow light were encountered during the surgical resections.

### Extent of resection

GTR was achieved in 24 cases out of 39 patients scheduled for tumor removal (61.54%), in five cases a minimal residual volume was highlighted by postoperative MRI despite the intraoperative subjective evaluation, both under white-light and YELLOW 560 filter activation, of complete tumor removal (12.82%); furthermore, neuronavigation check at the end of the surgery did not reveal tumor remnants, probably due to the intrinsic limitation of this tool in cases of brain shift at the end of surgery. In the other 10 cases, the resection was subtotal with fluorescent residual spots to avoid neurological worsening (expected STR in 25.64%); three patients with rapidly worsening of clinical status were scheduled for a partial removal with debulking aim of large and hemorrhagic tumors to relieve functional structures (**Table 1**).

A young patient with an atypical spinal lesion was scheduled for open biopsy. The pathological spinal cord appeared enlarged and pale with a few, moderate fluorescent spots; with the aid of intraoperative neurological monitoring and contrast-enhanced ultrasound, these components were removed for histological analysis, which was diagnostic for pilocytic astrocytoma; the patient did not present any neurological sequelae. Finally, an open biopsy was performed for the left frontal tumor.

### Side effects and outcome

No adverse drug reaction related to SF injection was reported in this cohort of patients; the only remarkable and visible effect was the transient yellowish staining of urine, which disappeared in about 24 h.

At baseline, 30/44 (68.2%) of patients had KPS/LPS scores of 80–100, indicating good clinical and neurological conditions. Surgical morbidity led to a postoperative decline in KPS/LPS score in seven patients at discharge, but only in one case was the clinical worsening marked with a decrease of 40 points in the KPS/LPS score; 25 patients were discharged with an unchanged KPS/LPS score, whereas 12 patients presented with a clinical improvement using surgical treatment. At discharge, 31/44 (70.5%) of patients had KPS/LPS scores of 80–100 (**Table 1**).

Long-term follow-up data were available for 39 patients; all of them were alive during this time; the follow-up period ranged between 2 and 58 months, with a median follow-up of 26.4 months. Most patients (33/39—84.6%) were neuroradiologically stable (tumor free or stable remnant tumor); 15 patients presented a clinical improvement measured using the KPS/LPS score, 17 patients were unchanged and only one patient presented with a slight clinical worsening. Six patients presented with tumor recurrency or progression disease, but only two were clinically worsened; two of them were scheduled for re-do surgery, whereas the other two were for adjuvant radiotherapy and chemotherapy. Two patients are prosecuted for follow-up with a *wait and see* approach (**Table 1**).

## Discussion

To our knowledge, this is the first report specifically evaluating the use of fluorescein-guided surgery in PA resection. Given that the contrast uptake in the preoperative T1-weighted MRI reflects the fluorescent staining pattern of the tumor (14, 20), we hypothesized that the use of the SF would have positively affected the intraoperative magnification of these neoplasms. Indeed, the use of SF could improve tumor visualization and, therefore, the entity of tumor removal (19); our observation was associated with contrast enhancement on preoperative MRI, which was present in almost all tumors in our series.

Thirty-nine patients enrolled in the study were scheduled for surgery with a previous planning of macroscopic resection but keeping in mind the philosophy of maximal safe resection: in this series, we obtained GTR in more than half of the patients (24/39, 61.54%); these data can be explained by the fact that many tumors were located in such eloquent regions in which a sub-total resection is sometimes advisable. As a matter of fact, minimal residual tumor (lower than 10% of preoperative tumor volume) at postoperative MRI was expected in 10 cases (25.64%), as it was involving eloquent areas or due to the adherence to major brain vessels or cranial nerves and was therefore independent of the fluorescence visualization. Conversely, in five cases (12.82%), the residual tumor was an unexpected finding, based on the absence of clear residual intraoperative fluorescent tissue; this can be related, as for other fluorophores, to the fact that the residual tissue was not exposed during resection because it was hidden under the normal brain parenchyma. In almost all cases, surgeon-reported visibility of pathological tissue was clearly enhanced by SF and YELLOW 560 filter views and was almost always judged helpful for complete tumor resection.

Pilocytic astrocytomas represent 1.6% of all CNS neoplasms and account for 5% of all glial tumors annually diagnosed in children and adults (1, 5, 8); approximately 60% of PAs are diagnosed in the pediatric population, representing about 17% of all intracranial tumors in children (30). They can arise anywhere along the neuroaxis, but they are usually found in deep midline structures such as the cerebellum, the opticochiasmatic region and hypothalamus, the brainstem, and the spinal cord (4). In the pediatric population, the cerebellum is the most common site of presentation (2, 9); In contrast, no difference in tumor distribution was found in adults between the infratentorial and supratentorial compartments (8, 31).

According to the WHO CNS5 classification (7), pilocytic astrocytomas are considered circumscribed astrocytic gliomas in consideration of their more solid growth pattern, as opposed to the strongly infiltrative behavior of diffuse astrocytomas. Indeed, PAs usually present an overall indolent course in the pediatric population (2, 32); unfavorable prognostic factors negatively affecting the overall survival (OS) are the diencephalic region location, the pilomyxoid variant, the proliferation index, or the adherence and infiltration of the thick cystic wall to the brain, prejudicing surgical radicality (11). Aggressive surgical resection continues to be one of the most important prognostic factors (32, 33) because of its irrefutable correlation with both overall survival and progression-free survival (PFS).

According to recent published data by different international groups (10, 34–38), GTR was achievable in about half of the patients operated with white-light illumination (53.81%). In this perspective, the application of new technical tools aimed at improving the EOR could be beneficial in the management of PAs. During the last few years SF has emerged as an intraoperative tracer able to improve brain–tumor visualization (19–21), due to its vascular, non-specific mechanism of action related to the accumulation in brain regions with BBB disruption (14, 17); these areas present altered permeability that seems to correlate consistently with the contrast-enhanced portions of T1-weighted MRI sequences, accounting for the staining capacity of this fluorescent tracer with tumors that uptake contrast (26). Previous experiences suggested that the use of SF could be associated with a bright fluorescence of the tumor area in primary and recurrent high-grade gliomas (19, 20, 25, 39), in brain metastases (22), in primary CNS lymphomas (23), and in gangliogliomas (21). This was also associated with good results in terms of the extent of resection as well as PFS and OS. Use of the fluorophore 5-aminolevulinic acid (5-ALA), a biochemical precursor of hemoglobin that provokes the synthesis and accumulation of fluorescent porphyrins in different tumors, has been reported but with inconsistent results: in a recent systematic review by Stummer and coworkers (40), in which 5-ALA was evaluated in different pediatric tumors, including 33 PAs, strong fluorescence could only be detected in four cases, therefore accounting for a judgment of usefulness only in 12.1% of the entire series.

Additionally, the recent introduction of a dedicated filter integrated into the surgical microscope, specific for the excitation and emission wavelength of sodium fluorescein, has further improved the intraoperative visualization of the tumor and the surrounding brain parenchyma (16, 17). This technological adjunct significantly affected the development of fluorescein-guided surgery, having increased the discrimination of tumoral tissue from the surrounding viable brain parenchyma. Our results seem to suggest that fluorescein-guidance for surgical resection may improve the intraoperative discrimination of the tumor margin and potentially increase the GTR rate.

Although intraoperative evaluation of the fluorescence was subjective in this study, the report of helpful fluorescence in almost all cases, particularly at the tumor margins, taken together with the rate of GTR, suggests a reproducible effect. Fluorescein is still undergoing feasibility tests, especially along with the specific YELLOW 560 filter. It is important to stress that, in most countries, the use of SF as a tracer in neuro-oncology should still be considered off-label; thus, the widespread use of fluorescence-guided surgery will depend on the definitive approval by the competent authorities (17, 20). Optimal dosage and timing of SF in PA surgery is neither known nor experienced in this study. Also, in this cohort of tumors, we have used a low dosage of 5 mg/kg, intravenously injected at the time of patient intubation (16), as originally proposed by our group in January 2012 (25).

The main limitation of the presented study is represented by the lack of data about OS; given the prolonged survival of PAs, a long-term follow-up is not available in our data; for this reason, along with the inclusion of both primary and recurrent PAs, no proper patient evaluation from a prognostic perspective was possible. Moreover, we did not make any comparison between the use of SF and white-light illumination or other available fluorophores, like 5-ALA. Despite these weaknesses, our study could represent a proof of concept that may indicate that fluorescein use is potentially useful in the identification of tumoral tissue and in achieving a high rate of GTR of PAs. As previously demonstrated with other lesions, further studies could better elucidate the contribution of fluorescein-guided technique in improving the intraoperative visualization during surgical resection of this tumor subtype. Furthermore, the effect of fluorescein on PFS and OS needs to be evaluated in a randomized, controlled clinical trial with adequate power to precisely assess the outcomes within a predefined observation period.

## Conclusion

Our data suggest a positive effect of fluorescein-guided surgery during resection of PAs with contrast enhancement on preoperative MRI. We assert that SF is a safe and feasible tool; the use of fluorescein and a YELLOW 560 filter is a readily available method for fluorescence-guided tumor resection, allowing intraoperative tumor visualization similarly to contrast enhancement in brain MRI.

## Data availability statement

The raw data supporting the conclusions of this article will be made available by the authors, without undue reservation.

## Ethics statement

The studies involving human participants were reviewed and approved by the Ethical Committees of the Fondazione IRCCS Istituto Neurologico Carlo Besta and of the University Medical Center Regensburg. Written informed consent to participate in this study as well as for the publication of any potentially identifiable images or data included in this article was obtained from the individual(s), and minor(s)’ legal guardian/next of kin.

## Author contributions

JF and FA: study concept and design. JF, JH, K-MS, and FA: critical revision of the manuscript for intellectual content. All authors: acquisition of data, data analysis and interpretation. K-MS, PF and FA: study supervision. All authors listed have made a substantial, direct, and intellectual contribution to the work and approved it for publication.

## Funding

This study was partially supported by the Associazione Paolo Zorzi per le Neuroscienze, ONLUS and by the Italian Ministry of Health (RRC).

## Conflict of interest

FA, JH, and K-MS received honoraria from the Carl Zeiss Meditec Company for lectures at International Congresses.

The remaining authors declare that the research was conducted in the absence of any commercial or financial relationship that could be constructed as a potential conflict of interest.

## Publisher’s note

All claims expressed in this article are solely those of the authors and do not necessarily represent those of their affiliated organizations, or those of the publisher, the editors and the reviewers. Any product that may be evaluated in this article, or claim that may be made by its manufacturer, is not guaranteed or endorsed by the publisher.
